# ^13^C-assisted metabolic flux analysis to investigate heterotrophic and mixotrophic metabolism in *Cupriavidus necator* H16

**DOI:** 10.1007/s11306-017-1302-z

**Published:** 2017-12-04

**Authors:** Swathi Alagesan, Nigel P. Minton, Naglis Malys

**Affiliations:** BBSRC/EPSRC Synthetic Biology Research Centre (SBRC), School of Life Sciences, Centre for Biomolecular Sciences, University Park, The University of Nottingham, Nottingham, NG7 2RD UK

**Keywords:** *Cupriavidus necator* H16, Metabolic flux analysis, ^13^C-labelling, Steady-state, Amino acids, RT-PCR

## Abstract

**Introduction:**

*Cupriavidus necator* H16 is a gram-negative bacterium, capable of lithoautotrophic growth by utilizing hydrogen as an energy source and fixing carbon dioxide (CO_2_) through Calvin–Benson–Bassham (CBB) cycle. The potential to utilize synthesis gas (Syngas) and the prospects of rerouting carbon from polyhydroxybutyrate synthesis to value-added compounds makes *C. necator* an excellent chassis for industrial application.

**Objectives:**

In the context of lack of sufficient quantitative information of the metabolic pathways and to advance in rational metabolic engineering for optimized product synthesis in *C. necator* H16, we carried out a metabolic flux analysis based on steady-state ^13^C-labelling.

**Methods:**

In this study, steady-state carbon labelling experiments, using either d-[1-^13^C]fructose or [1,2-^13^C]glycerol, were undertaken to investigate the carbon flux through the central carbon metabolism in *C. necator* H16 under heterotrophic and mixotrophic growth conditions, respectively.

**Results:**

We found that the CBB cycle is active even under heterotrophic condition, and growth is indeed mixotrophic. While Entner–Doudoroff (ED) pathway is shown to be the major route for sugar degradation, tricarboxylic acid (TCA) cycle is highly active in mixotrophic condition. Enhanced flux is observed in reductive pentose phosphate pathway (redPPP) under the mixotrophic condition to supplement the precursor requirement for CBB cycle. The flux distribution was compared to the mRNA abundance of genes encoding enzymes involved in key enzymatic reactions of the central carbon metabolism.

**Conclusion:**

This study leads the way to establishing ^13^C-based quantitative fluxomics for rational pathway engineering in *C. necator* H16.

**Electronic supplementary material:**

The online version of this article (10.1007/s11306-017-1302-z) contains supplementary material, which is available to authorized users.

## Introduction

*Cupriavidus necator* H16 (also known as *Ralstonia eutropha* H16) is a Gram-negative bacterium that belongs to the order *Burkholderiales*, class *Betaproteobacteria*. H16 has been isolated from a soil near Goettingen, Germany, almost 60 years ago (Schlegel et al. 1961a, b; Wilde 1962). Since then, it has become the most-studied hydrogen-oxidising ‘Knallgas’ bacterium with best-characterised poly(3-hydroxybutyrate) (PHB) metabolism.

*Cupriavidus necator* H16 is a facultative anaerobe, which can switch to anaerobic respiration by using NO_3_ and NO_2_ as electron acceptors. It grows utilizing a variety of organic substrates ranging from sugars, fatty acids, amino acids and citric acid cycle intermediates. It is also capable to fix directly a carbon dioxide (CO_2_) through the CBB cycle using hydrogen as the energy source (Bowien and Kusian 2002), making it a useful organism for studying mixotrophic and autotrophic metabolism. Under unbalanced nutrient conditions the organism produces and stores large quantities of PHB (Steinbüchel and Füchtenbusch 1998). The ability to utilize a range of organic and inorganic substrates, and possibility of re-routing the stored carbon to valuable products makes *C. necator* an excellent chassis to develop as a “single cell factory”.

The genome of *C. necator* H16 is composed of chromosomes 1 (4.1 Mbp) and 2 (2.9 Mbp), and a megaplasmid pHG1 (0.45 Mbp). The sequence for the megaplasmid was published in 2003 while the whole genome was reported in 2006 (Pohlmann et al. 2006; Schwartz et al. 2003). Chromosome 1 mainly codes for essential genes while most genes for alternative metabolism are located on chromosome 2 (Pohlmann et al. 2006; Schwartz et al. 2009). Genes for the CBB cycle are found in two operons localized on the chromosome 2 and the megaplasmid (Bowien and Kusian 2002). The latter thought to be acquired recently by the organism (Schwartz et al. 2009). A large proportion of the genome encodes for transport genes, which explains the wide range of substrates the organism can utilize for growth (Schwartz et al. 2009).

In order to develop *C. necator* as a chassis for producing platform chemicals from organic wastes like glycerol or inorganic substrates such as CO_2_, it is imperative to have in-depth knowledge of the underlying metabolism. Genome-wide transcriptome (Brigham et al. 2010; Peplinski et al. 2010; Shimizu et al. 2013), and proteome analysis (Schwartz et al. 2009) have been undertaken to study the metabolism under different growth conditions. A majority of metabolomics studies have been mainly focused on poly-3-hydroxybutyrate (PHB) production in *C. necator*. In particular, a genome scale reconstruction and in silico analysis for PHB synthesis has shown that efficient PHB synthesis can only occur under condition if carbon/nitrogen uptake ratio is up to 7.5 and it decreases when carbon/nitrogen ratio becomes higher and growth rate is maximized (Park et al. 2011). Despite availability of metabolite profiles under different growth phases on fructose and octanoate (Fukui et al. 2014), as well as the network analysis and mathematical modelling to provide better understanding about the metabolism (Lopar et al. 2014), there is a lack of more realistic predictions of underlying reaction rates that are directly obtained through experimental studies. Furthermore, only limited experimental data for lithoautotrophic metabolism is available.

^13^C-assisted metabolic flux analysis is often used for quantitative characterisation of the metabolism providing additional constrains to flux analysis prediction and more accurate insight into the distribution of flux through metabolic pathways (Wiechert and de Graaf 1997; Wiechert et al. 1997; Wittmann 2007). In the steady-state ^13^C-metabolic flux analysis (^13^C-MFA), the labelling profiles of proteinogenic amino acids are traced back through the pathways to the substrates allowing to estimate the fluxes through the various pathways and providing a true representation of the metabolic state under studied condition (Zamboni et al. 2009). A number of analysis tools such as Fiatflux, OpenFLUX, 13CFLUX2, iMS2Flux are available for estimating the carbon flux from steady-state ^13^C-labeling information (Poskar et al. 2012; Quek et al. 2009; Weitzel et al. 2013; Zamboni et al. 2005).

To investigate how change between heterotrophic and autotrophic metabolisms may affect carbon flux we performed steady-state ^13^C-MFA of the central carbon metabolism of *C. necator* H16 under heterotrophic growth with ^13^C-labelled fructose or ^13^C-labelled glycerol, and mixotrophic growth with ^13^C-labelled glycerol and CO_2_. Since steady-state ^13^C-MFA with a single carbon substrate such as CO_2_ will not result in differential labelling, the carbon fixation by CBB cycle was studied under mixotrophic growth condition. This is the first study on carbon labelling experiments for *C. necator*, which provides qualitative and quantitative information on carbon flux distribution under the different growth conditions.

## Materials and methods

### Culture and growth conditions

Axenic cultures of wild-type *C. necator* H16 were grown and maintained in Luria–Bertani medium, in an orbital shaker incubator at 200 rpm and 30 °C. For heterotrophic growth, cultures were grown in minimal medium (Schlegel et al. 1961a) supplemented with 4 g L^−1^ fructose or 10 g L^−1^ glycerol, and the growth was monitored by measuring the optical density at 600 nm. For mixotrophic cultivation, the cultures were grown in 120 mL serum bottles with hydrogen, carbon dioxide and air in the ratio 8:1:1 (v/v/v) (Schwartz et al. 2009) in minimum medium supplemented with 10 g L^−1^ glycerol.

### Carbon labelling experiments

Carbon labelling studies were carried out with d-[1-^13^C]fructose and [1,2-^13^C]glycerol (both compounds with isotopic purity of 99 atom% ^13^C; Sigma-Aldrich, St. Louis, MO). In heterotrophic growth, medium was supplemented with 4 g L^−1^ d-[1-^13^C]fructose or 10 g L^−1^ [1,2-^13^C]glycerol; while in mixotrophic growth, the serum bottles were filled with 10% CO_2_ and the medium was supplemented with 10 g L^−1^ [1,2-^13^C]glycerol. Cells were sub-cultured twice in medium containing ^13^C-substrate to minimize the effect of unlabelled initial biomass on the labelling profile of the amino acids. Cells in mid-exponential phase were used to inoculate 5 mL minimal medium with the labelled substrate to an initial OD_600_ = 0.01 and allowed to grow till OD_600_ = 1. This culture was used to inoculate fresh medium with labelled substrate to an initial OD_600_ = 0.01 and two sets of samples for GC-MS, RT-PCR and biochemical analysis were collected in the exponential phase at OD_600_ = 0.6 and OD_600_ = 1.2.

### GC-MS measurements

Culture volume equivalent to OD_600_ = 3 was centrifuged and the pellet was hydrolyzed with 700 µL of 6 N HCl at 100 °C for 16 h. The hydrolysis tube was centrifuged, the supernatant was transferred to fresh tube and evaporated to dryness on a heating block at 60 °C. Subsequently, 150 µL of ultra-pure water was added to fully dissolve the dried sample, which was filtered through a 0.2 µm syringe filter and again evaporated to dryness on a heating block at 60 °C. The dried sample was dissolved in 50 µL anhydrous pyridine, followed by derivatisation with 70 µL MTBSTFA + 1% TBDMCS (Sigma-Aldrich, St. Louis, MO) for 30 min on a heating block at 60 °C. The samples were centrifuged, and the supernatant was transferred to GC vials for analysis (Young et al. 2013).

The derivatized amino acids were analyzed using Agilent 6890N GC fitted with Agilent 5973N MSD equipped with 30 m × 0.25 mm × 0.25 µm Agilent HP-5MS column (Agilent, California, United States). The inlet and interface temperatures were maintained at 270 and 300 °C, respectively. The oven temperature was set at 150 °C for 2 min, then increased at a rate of 5 °C/min to 280 °C, and finally held at 280 °C for 2 min with total run time of 30 min (Young et al. 2013). Hydrogen was used as the carrier gas at a flow rate of 0.8 mL min^−1^. 1 µL of the derivatized sample was injected with a split flow set at 10:1. The quadrupole MS detector was operated in electron impact (EI) ionization mode with ion source temperature and quadrupole temperatures set at 230 and 150 °C, respectively, with a full scan detection (100–500 m/z).

### Flux analysis

IsoCor software was used to correct the factional labelling distribution of the amino acids for natural isotopic abundance (Millard et al. 2012). The corrected MIDs (mass isotopomer distribution) was used for flux analysis using OpenFLUX software as previously described (Quek et al. 2009). The metabolic model for central carbon metabolism was derived from published genome scale model of *C. necator* H16 and carbon atom transitions were adopted from literature reports (Alagesan et al. 2013; Park et al. 2011; Stryer 1995).

The summed fractional labelling (SFL) (Eq. 1) gives the fractional labelling of the carbon positions in the amino acid fragment (i.e. a measure for the average number of ^13^C-atoms in the amino acid fragment) (Christensen and Nielsen 2000), and is calculated by:

1 $$SFL=\frac{{\varepsilon i \times {M_i}}}{{\varepsilon {M_i}}}$$

where *M*_*i*_ is the corrected mass isotopomer fraction with *i* ^13^C-atoms.

The R-value, which is the ratio of the flux from [1,2-^13^C]glycerol (*V*_*gly*_, 99% purity) to the flux from unlabeled ^12^CO_2_ (*V*CO_2_, 1% natural ^13^C abundance), was calculated using Eq. 2 (Feng et al. 2010),

2 $$\frac{{0.99 \times n \times {V_{gly}}+~0.01 \times {V_{{\text{c}}{{\text{o}}_2}}}}}{{m \times {V_{gly}}+~{V_{{\text{c}}{{\text{o}}_2}}}}}=~\frac{{\sum\nolimits_{{i=1}}^{C} {i\times{M_i}} }}{C} \Rightarrow R=\frac{{{V_{gly}}}}{{{V_{{\text{c}}{{\text{o}}_2}}}}}$$

where n = 2 is the number of ^13^C-labelled carbon in glycerol and m = 3 is the total number of carbon atoms in glycerol. *M*_*i*_ gives the isotopomer fraction of the amino acid fragment with “i” ^13^C carbon atoms and C is the total number of carbon atoms in the amino acid backbone.

### Biochemical analysis

The high performance liquid chromatography (HPLC) in combination with ultraviolet spectroscopy (UV) and refractive index (RI) detection was used for quantitative analysis of d-fructose and glycerol, respectively. The concentrations of these compounds in the supernatant were estimated from standard curves generated by analysing known concentrations of d-fructose and glycerol (both ≥ 99% purity; Sigma-Aldrich). Routinely, samples were diluted 1:1 with the mobile phase (5 mM sulphuric acid) containing 50 mM valerate and filtered using a 0.2 µm syringe filter. 20 µL of the sample was injected into Dionex UltiMate 3000 HPLC system (Thermo Scientific, Waltham, Massachusetts) fitted with ERC RefractoMax520 RI detector and a UV-DAD at 210 nm. The sample components were separated using Aminex HPX-87H (Bio-Rad, Hercules, CA) using 5 mM sulphuric acid as the mobile phase at a flow rate of 0.5 mL min^−1^.

### Real time PCR

Culture volume equivalent to OD_600_ = 2 was collected, 1 ml of TRI reagent (Sigma Aldrich, St. Louis, MO) was added to the pellet and samples were stored at − 80 °C. The total RNA from the cell pellet was extracted using manufacturer’s recommendation. The RNA samples were DNase treated using RQ1 DNase kit (Promega, Madison, Wisconsin) followed by cDNA synthesis of 2 µg sample using ProtoScript^®^ II First Strand cDNA Synthesis Kit (New England Biolabs, Ipswich, Massachusetts). Quantitative real time-PCR was carried out Light cycler 480 II (Roche Diagnostics, Indianapolis, IN) using LuminoCt^®^ SYBR^®^ Green qPCR ReadyMix™ (Sigma-Aldrich, St. Louis, MO) and gene specific primers. The expression of genes was normalized to the expression of 16S rRNA, which was used as the internal control. No template control reactions were set-up for each primer pair. The list of oligonucleotide primers used for RT-PCR is provided in Supplementary Table S1.

## Results

In order to understand the metabolism of organic and inorganic carbon substrates by *C. necator* H16, heterotrophic growth in^13^C-labelled fructose and glycerol was studied, whereas the lithoautotrophic metabolism was investigated using a mixotrophic growth condition with ^13^C-glycerol and unlabelled CO_2_. Analysis of culture growth profile showed that the growth rate was highest under heterotrophic condition with fructose, while it was slowest for heterotrophic growth with glycerol (Table 1), complementing previous studies (Friedrich et al. 1981; Lopar et al. 2014). The slow growth with glycerol is attributed to the reactive oxygen species (ROS) produced by the activity of hydrogenases in this condition (Schwartz et al. 2009).

**Table 1:** Specific growth rates and substrate uptake rates for *C. necator* H16 grown under heterotrophic condition with d-[1-^13^C]fructose, [1,2-^13^C]glycerol and under mixotrophic condition with [1,2-^13^C]glycerol and unlabelled CO_2_

	Specific growth rate (h^−1^)	Doubling time (h)	Substrate uptake rate (g L^−1^ (OD)^−1^ h^−1^)
Heterotrophic growth			
d-[1-^13^C]fructose	0.254	2.7	0.189
[1,2-^13^C]glycerol	0.173	4	0.023
Mixotrophic growth			
[1,2-^13^C]glycerol	0.23	3	0.055

### Mass isotopomer distribution

**Fig. 1:**
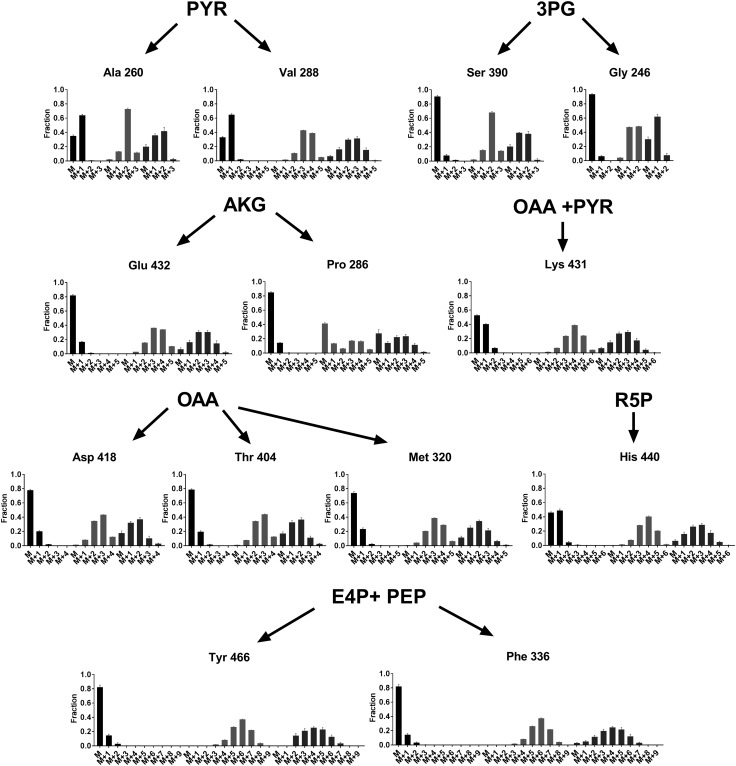
The corrected mass isotopomer distribution (MID) of [M-57] fragment ions of TBDMCS-derivatized amino acids in the isotopic steady state under heterotrophic growth with d-[1-^13^C]fructose (black bars) or [1,2-^13^C]glycerol (red bars) and under mixotrophic growth with [1,2-^13^C]glycerol and unlabelled CO_2_ (blue bars). The y-axis represent the number of ^13^C atoms incorporated into the amino acid molecule, increasing the m/z value by M + 1, M + 2 and so on. The MID of alanine, valine, serine, glycine, glutamate, proline, tyrosine, phenylalanine, aspartate, threonine, methionine, lysine, and histidine is mapped to their respective precursor molecules pyruvate (PYR), 3-phosphoglycerate (3-PG), alpha-ketoglutarate (AKG), erythrose-4phosphate (E4P), phosphoenolpyruvate (PEP), oxaloacetate (OAA) and ribose-5-phosphate (R5P)

^13^C-assisted metabolic flux analysis uses differential ^13^C-labelling patterns of proteinogenic amino acids for metabolic network-wide isotope balancing and flux estimation. In order to establish amino acids labelling patterns under heterotrophic and mixotrophic growth conditions, ^13^C-labelling experiments were performed using either d-[1-^13^C]fructose or [1,2-^13^C]glycerol, and mixture of [1,2-^13^C]glycerol with CO_2_, respectively, as described in “Material and methods”. The amino acid fractional labelling was corrected for natural isotope abundance to generate the mass isotopomer distribution (MID) values (Supplementary Tables S4, S5, S6). The fragmentation profiles of 15 amino acids were analysed. Figure 1 and Supplementary Fig. S1 provides a graphical representation of the MIDs of the different amino acid fragments (m/z corresponding to [M-57] and [M-85], respectively) under the different growth conditions mapped to their precursor metabolite. Amino acid fragments produced by the same precursors showed similar labelling pattern across different substrates, except the labelling profile of proline (M_0_ = 286) under heterotrophic and mixotrophic growth with glycerol did not complement that of glutamate (M_0_ = 432), due to its co-elution with another compound resulting in inaccurate MID (Antoniewicz et al. 2007). Reduced label incorporation was observed in cultures grown with d-[1-^13^C]fructose, while the distribution is skewed to higher mass isotopomers with [1,2-^13^C]glycerol, which is also observed in the summed fractional labelling (SFL) values (Table 2). The SFL of the amino acids under mixotrophic condition is lower than under heterotrophic condition, suggesting dilution of ^13^C by unlabelled CO_2_ fixed through the CBB cycle. R-value gives the ratio of carbon flux from labelled glycerol to that from unlabelled CO_2_ and is a measure of the extent of mixotrophic growth. The R-values in the present study for the amino acids were in the range of 0.4–0.8 (Table 2).

**Table 2:** Summed fractional labelling (SFL) for [M-57] fragment ions of amino acids under the three different growth conditions and the R-value for amino acids under mixotrophic growth

	Heterotrophic growth		Mixotrophic growth	
	SFL	SFL	SFL	R-value
	Fructose	Glycerol	Glycerol + CO_2_	Glycerol + CO_2_
Ala (260)	0.658	1.941	1.265	0.576
Gly (246)	0.065	1.440	0.776	0.463
Val (288)	0.689	3.343	2.353	0.810
Met (320)	0.291	3.112	1.891	0.436
Ser (390)	0.108	1.946	1.213	0.514
Thr (404)	0.233	2.597	1.494	0.423
Phe (336)	0.217	5.809	3.882	0.614
Asp (418)	0.244	2.574	1.486	0.418
Glu (432)	0.194	3.329	2.363	0.823
Tyr (466)	0.209	5.809	4.089	0.565
His (440)	0.599	3.757	2.505	0.720

### Steady state metabolic flux analysis

**Fig. 2:**
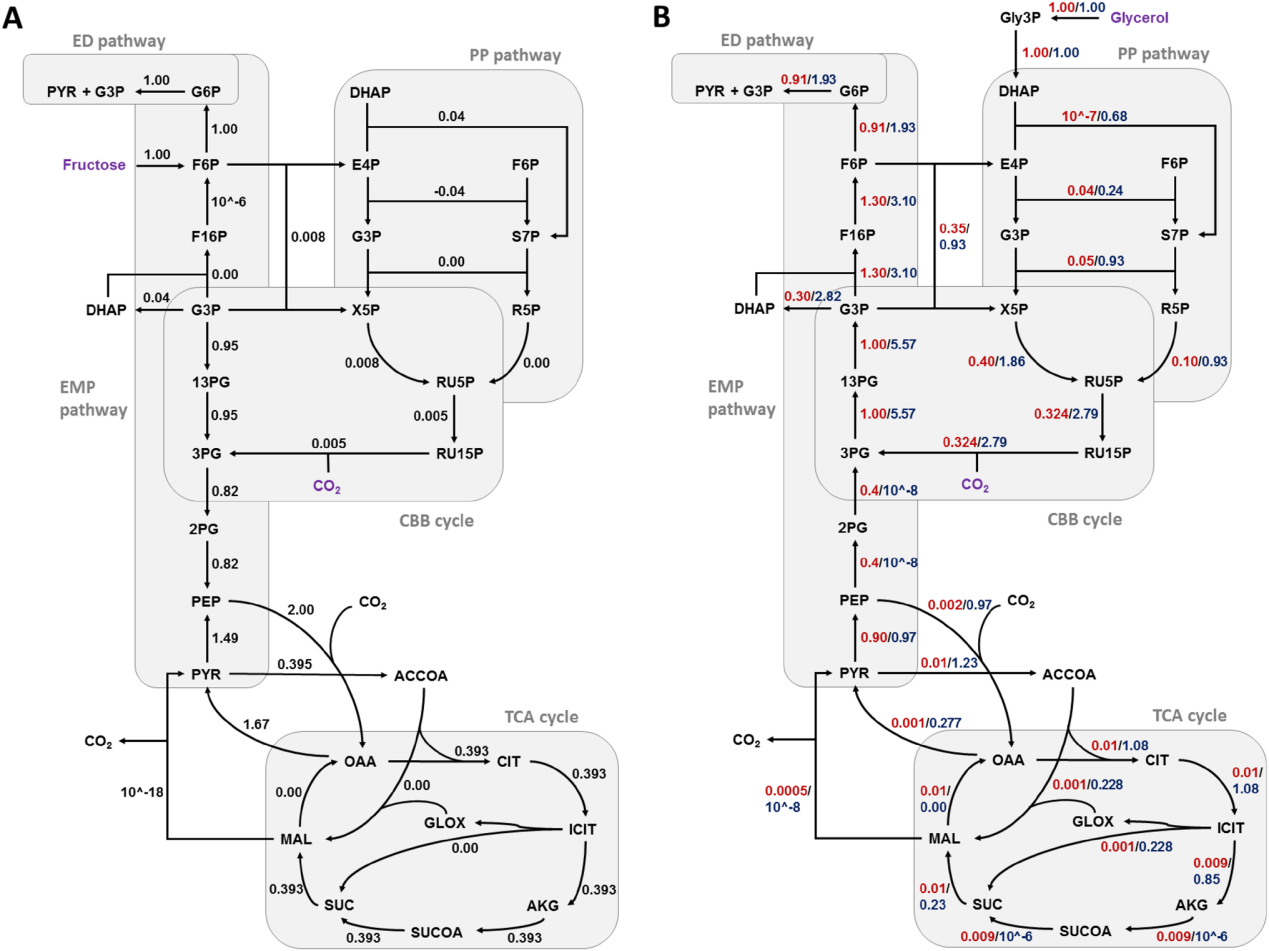
The steady-state intracellular reaction rates predicted using ^13^C-amino acids labelling profiles. The steady-state reaction rates in the central carbon metabolism of *C. necator* H16 are shown in black for heterotrophic growth with d-[1-^13^C]fructose (**a**), and in red or blue for heterotrophic growth with [1,2-^13^C]glycerol or mixotrophic growth with [1,2-^13^C]glycerol and unlabelled CO_2_ (**b**). Metabolite abbreviations: *Gly3P* glycerol-3-phophate, *DHAP* dihydroxyacetone phosphate, *E4P* erythrose-4-phosphate, *F6P* fructose-6-phosphate, *G3P* glyceraldehyde-3-phospahte, *S7P* sedoheptulose-7-phosphate, *X5P* xylulose-5-phosphate, *R5P* ribose-5-phosphate, *RU5P* ribulose-5-phosphate, *RU15P* ribulose-1,5-bisphosphate, *G6P* glucose-6-phosphate, *F16P* fructose-1,6-phosphate, *13PG* 1,3-phosphoglycerate, *3-PG* 3-phosphoglycerate, *2-PG* 2-phosphoglycerate, *PEP* phosphoenolpyruvate, *PYR* pyruvate, *ACCOA* acetyl-CoA, *CIT* citrate, *ICIT* isocitrate, *AKG* alpha-ketoglutarate, *SUCOA* succinyl-coA, *SUC* succinate, *MAL* malate, *OAA* oxaloacetate, *GLOX* glyoxylate

Of the 15 amino acids detected, the labelling information of 12 amino acids ([M-57 and M-85]) was used for flux analysis (Fig. 1 and Supplementary Fig. S1). MIDs of tyrosine and lysine were not used due to their low signal to noise ratio, while that of proline was avoided due to measurement inaccuracy resulting from co-elution with another compound (Antoniewicz et al. 2007). OpenFLUX software was used to compute the fluxes through the 72 pathway reactions of the central carbon metabolism (Quek et al. 2009) (Supplementary Table S2). Predicted flux estimates were the converged results from 100 iterations of the optimization algorithm (Fig. 2). The minimum residual error was 498, 259 and 265 for the simulations under heterotrophic growth with fructose, with glycerol and mixotrophic growth, respectively (Supplementary Fig. S2). The 95% confidence interval for the estimates was calculated to determine the accuracy of the predictions (Supplementary Table S3).

The ribulose-1,5-bisphosphate carboxylase/oxygenase (RuBisCO) activity and other CBB cycle enzymes have been shown to be present at substantial levels in cells during heterotrophic growth with fructose and succinate (Friedrich et al. 1981; Schwartz et al. 2009). In addition to the CBB cycle enzymes, elevated activity is noted for hydrogenase in *C. necator* cells utilizing glycerol as the carbon substrate (Friedrich et al. 1981). Therefore, the CBB cycle was included in the model for flux distribution simulations even under heterotrophic growth condition. Whereas, a very low flux was observed through CBB cycle under heterotrophic growth with fructose, significant flux was predicted with glycerol, suggesting that the growth in this condition was not solely heterotrophic (Fig. 2). In the latter condition, CO_2_ can become available from the oxidative decarboxylation of l-malate (hereafter denoted malate) by MaeB. This observation is supported by the predicted increase in the flux from malate to pyruvate (Fig. 2a). Eight-fold higher flux is observed through CBB cycle under mixotrophic condition possibly due to higher availability of CO_2_ in this condition (Fig. 2b). It is interesting to note the partitioning of flux through the glyoxylate pathway and TCA cycle in the different growth conditions, which is important for maintaining a balance between energy production and precursor synthesis (Walsh and Koshland 1984).

### Gene expression studies

**Fig. 3:**
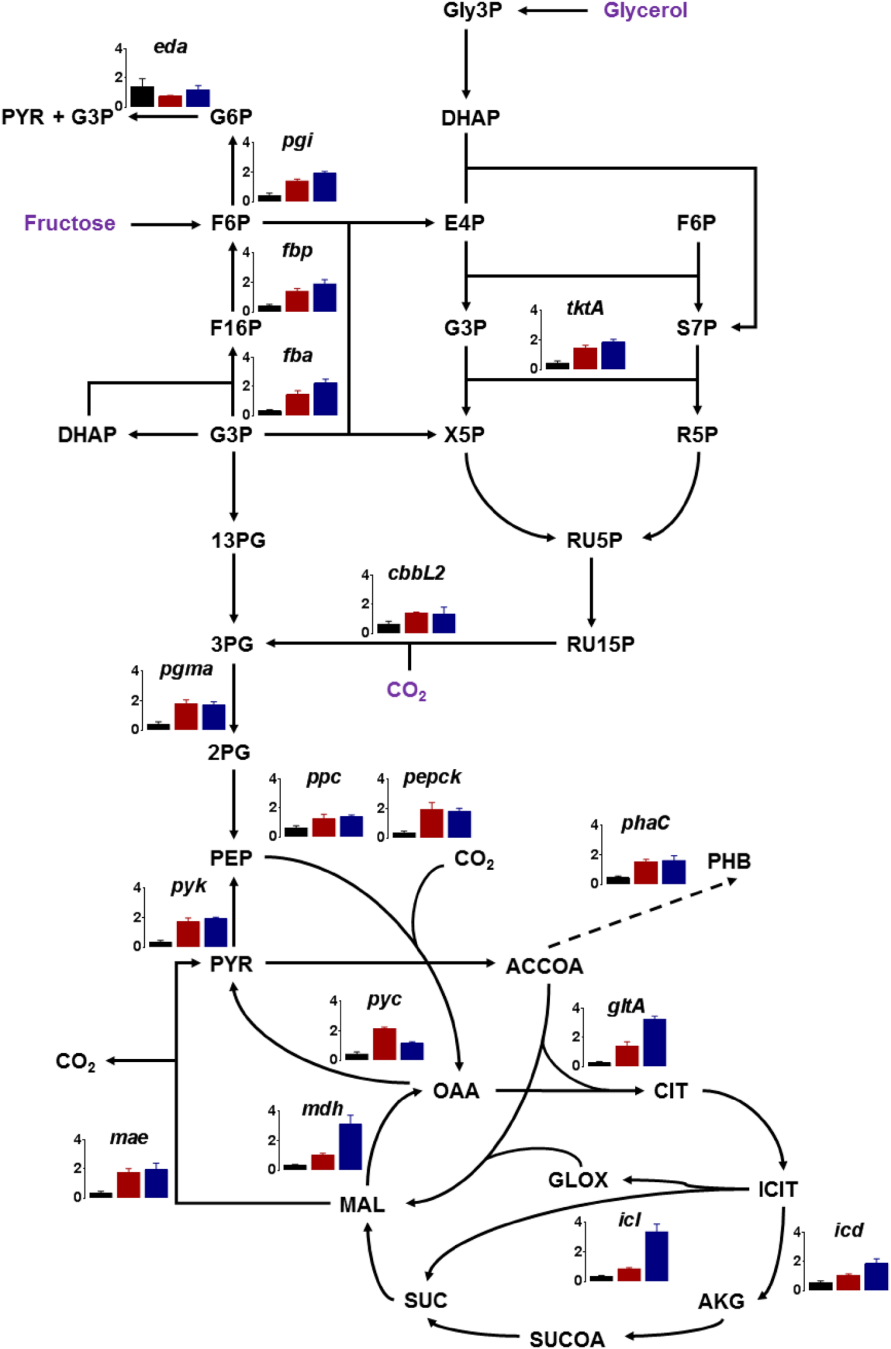
Graphical representation of the relative mRNA levels of selected genes measured using real time PCR under heterotrophic growth with d-fructose (black bars) or glycerol (red bars) and under mixotrophic growth with glycerol and CO_2_ (blue bars). Genes analysed: *tktA* transketolase, *fbp* fructose bisphosphatase, *rbcL* ribulose-1,5 bisphosphate carboxylase/oxygenase, large subunit, *pyk* pyruvate kinase, *gltA* citrate-synthase, *icd3* isocitrate dehydrogenase, *mdh* malate dehydrogenase, *pyc* pyruvate carboxylase, *maeB* malate decarboxylase, *ppc* PEP carboxylase, *pck* PEP carboxykinase, *icl* isocitrate lyase, *pgi* phosphoglucose isomerase, *pgmA* phosphoglycerate mutase, *phaC1* polyhydroxyalkanoate synthase, *eda* 2-keto-3-deoxyphosphogluconate aldolase, *fba* fructose bisphosphate aldolase

The expression of 17 genes corresponding to critical reactions in the central carbon metabolism was studied using real time PCR to complement flux distribution results (Fig. 3). The expression level of all the genes except *eda* was much lower in fructose-grown cells than under heterotrophic and mixotrophic growth with glycerol conditions, possibly because ED pathway is the major sugar degradation route in *C. necator*. The transcript abundance of most genes is similar in glycerol grown cells under heterotrophic and mixotrophic condition with the exception of TCA cycle. The genes of the TCA cycle and glyoxylate pathway were highly induced under mixotrophic growth condition.

## Discussion

### Sugar metabolism

*Cupriavidus necator* H16 lacks genes encoding for phosphofructokinase of the Embden-Mayerhoff-Parnas (EMP) pathway and 6-phosphogluconate dehydrogenase of the oxidative pentose-phosphate (ox-PP) pathway (Pohlmann et al. 2006). Hence, the ED pathway is considered to be the major sugar degradation route. This is depicted in the carbon flux map (Fig. 2a), where fructose is converted to pyruvate and glyceraldehydes-3-phophate (G3P) by the ED pathway. Besides, RT-PCR data revealed that 2-keto-3-deoxy-6-phogluconate aldolase gene *eda*, encoding an enzyme that catalyses the conversion of 2-keto-3-deoxy-6-phosphogluconate (KDPG) to pyruvate and G3P, is induced under all three conditions studied, but the highest level of induction was observed under heterotrophic growth with fructose (Fig. 3). The latter finding is in agreement with previous results reported by Shimizu et al. (Shimizu et al. 2013). However, a twofold higher flux through the ED-pathway is predicted in mixotrophic condition. It is interesting to note that the predicted flux flows from G3P to PEP in heterotrophic growth with fructose, but the direction is reversed for growth with glycerol. This finding complements previous report suggesting that gluconeogenesis is the preferred metabolic route in glycerol-cultivated cells (Tanadchangsaeng and Yu 2012).

Pyruvate dehydrogenase complex catalyses the conversion of pyruvate to acetyl-CoA, is reportedly induced in heterotrophic growth condition (Shimizu et al. 2013). Acetyl-CoA is a major precursor for PHB synthesis. Notably, NADH and NADPH levels increase under nutrient limiting condition due to decrease in oxidation, causing inhibition of citrate synthase and isocitrate dehydrogenase in turn re-routing the carbon flux towards PHB production (Ienczak et al. 2011). In the present study, the carbon flux analysis revealed a enhanced flux towards acetyl-CoA under mixotrophic growth condition than that under heterotrophic growth with glycerol. The expression of *phaC1* gene, encoding a PHA synthase, is upregulated in cells grown under mixotrophic condition (Fig. 3) While insignificant flux (~ 10^−16^ mmol/g DW/h) is predicted towards PHB synthesis in fructose grown cells, which complements reports suggesting minimal PHB production in exponential growing cultures (Fukui et al. 2014; Raberg et al. 2008). The predicted flux, though marginal, is enhanced in glycerol grown cultures (~ 10^−7^ mmol/g DW/h and ~ 10^−3^ mmol/g DW/h in heterotrophic and mixotrophic conditions, respectively), complementing the transcript abundance profile.

### Pentose phosphate pathway

While the oxidative branch of the PPP pathway is incomplete due to the absence of 6-phosphogluconate dehydrogenase in *C. necator* (Pohlmann et al. 2006) the non-oxidative branch provides the precursors for nucleotides, aromatic amino acids and the CBB cycle. Low expression of transketolase gene *tktA* in fructose-grown cells is supported by the diminished flux predicted through non-oxidative PPP pathway. The higher reaction rate for non-oxidative PPP pathway under mixotrophic growth occurs possibly to supplement the precursor requirement for the CBB cycle (Fig. 2b). It is interesting to note that the flux analysis confirms an active CBB cycle for heterotrophic growth with glycerol. Significant activities and quantities of hydrogenases and CBB cycle enzymes have been reported previously in glycerol-grown cells (Friedrich et al. 1981; Schwartz et al. 2009). This supports hypothesis that the metabolism using glycerol as a carbon source even without additional supplementation of CO_2_ is indeed mixotrophic rather than heterotrophic(Shimizu et al. 2015). The dilution of label reflected by the SFL values (Table 2) and five-fold higher flux through CBB predicted in mixotrophic condition (Fig. 2b) is attributed to the enhanced CO_2_ fixation occurring in this condition, as mixotrophic growth was undertaken in 1:8 ratio of CO_2_:H_2_ in gas-tight bottles while heterotrophic growth occurred under ambient condition. Though RT-PCR results suggest expression of *cbbL2* in heterotrophic growth with fructose, similar to literature reports (Fukui et al. 2014; Shimizu et al. 2013), lower flux is predicted through CBB cycle possibly due to the low enzyme activity observed in this condition (Friedrich et al. 1981).

### Tricarboxylic acid cycle

Citrate synthase (gltA) catalyses the first reaction of the TCA cycle. The *gltA* gene is found to have the highest expression under mixotrophic condition (Fig. 3). A ~ twofold higher flux is observed under mixotrophic condition as compared to heterotrophic growth with fructose. However, it is interesting to note the distribution of flux through isocitrate lyase (Icl) and isocitrate dehydrogenase (Icd), which catalyse the conversion of isocitrate to glyoxylate and alpha-ketoglutrate (AKG), respectively. Majority of the carbon flux is directed to AKG in heterotrophic growth with fructose, which is also reflected in the higher transcript abundance for *icd* in this condition complementing previous observation (Shimizu et al. 2013). Though glyoxylate pathway is possibly active in heterotrophic condition with glycerol, ~ 25% of the carbon is diverted through the glyoxylate pathway in completely mixotrophic growth condition. Whereas it is suggested that glyoxylate pathway genes are highly expressed during growth on triacylglycerol (Brigham et al. 2010), isocitrate dehydrogenase protein concentration is reportedly 3.5 folds higher under lithoautotrophic condition (Schwartz et al. 2009).

The anaplerotic pathway involves inter-conversion of C3–C4 metabolites of glycolysis and TCA cycle. NADP-dependent malate decarboxylase (MaeB) catalyses the oxidative decarboxylation of malate to pyruvate, providing C3 metabolite and NADPH for anabolism (Bruland et al. 2010). The expression of *maeB* and malate dehydrogenase gene *mdh* is upregulated in trioleate-utilizing cultures (Brigham et al. 2010), which complements the flux in heterotrophic growth with glycerol. Flux is predicted through PEP carboxykinase (PEPCK) in glycerol-utilizing cultures complementing our RT-PCR results and literature reports, suggesting that the production of oxaloacetate (OAA) by carboxylation of phosphoenolpyruvate (PEP) is critical for the oxidative TCA cycle when it is running low (Tang et al. 2013).

### Energy metabolism

Carbon fixation by the CBB cycle is an energy intensive process requiring nine moles ATP and six moles NADPH per mole of 3-phosphoglycerate produced. In order to meet the energy demand for carbon fixation, pathways producing reducing energy equivalents are preferred. Highest Flux for CBB cycle is predicted under mixotrophic condition. This energy demand is possibly met by higher activity of NADPH producing pathways like isocitrate dehydrogenase, glucose-6-phosphate dehydrogenase, malate decarboxylase and glycerol-3-phosphate dehydrogenase, complemented by flux prediction (Fig. 2) and the higher glycerol uptake rate in mixotrophic condition (Table 1).

The present study provides the first qualitative and quantitative insight into the metabolism of *C. necator* H16 based on carbon labelling experiments. Similar experiments on mutant strains will provide knowledge for rationalized pathway engineering to direct the carbon flux towards product synthesis. For example in our present study we observed higher flux through the TCA cycle in mixotrophic growth condition which could be exploited for the production of platform chemicals like ethylene and itaconate, which are produced by TCA cycle intermediates. Furthermore, the production of ethylene from alpha-ketoglutarate could be enhanced by downregulating the glyoxylate pathway in mixotrophic condition. ^13^C-assisted dynamic flux analysis would be critical for studying lithoautotrophic metabolism and the flux distribution during PHB production, which is essential for re-routing the carbon flux from PHB production to product synthesis.

## Electronic supplementary material

Below is the link to the electronic supplementary material.

Supplementary material 1 (DOCX 319 KB)
