# Supplementary material for: 13C-assisted metabolic flux analysis to investigate heterotrophic and mixotrophic metabolism in Cupriavidus necator H16
Source: Metabolomics. 2017 Dec 4;14(1):9. doi: 10.1007/s11306-017-1302-z (PMC5715045; doi:10.1007/s11306-017-1302-z)
Supplement: Supplementary file 1 — Supplementary material 1 (DOCX 319 KB) [file 11306_2017_1302_MOESM1_ESM.docx]

**Supplementary Data**

^13^C-assisted metabolic flux analysis to investigate heterotrophic and mixotrophic metabolism in *Cupriavidus necator* H16

Swathi Alagesan, Nigel P. Minton, Naglis Malys*

BBSRC/EPSRC Synthetic Biology Research Centre (SBRC), School of Life Sciences, Centre for Biomolecular Sciences, University Park, The University of Nottingham, Nottingham, NG7 2RD, United Kingdom

*Author to whom correspondence should be addressed; e-mail: naglis.malys@nottingham.ac.uk


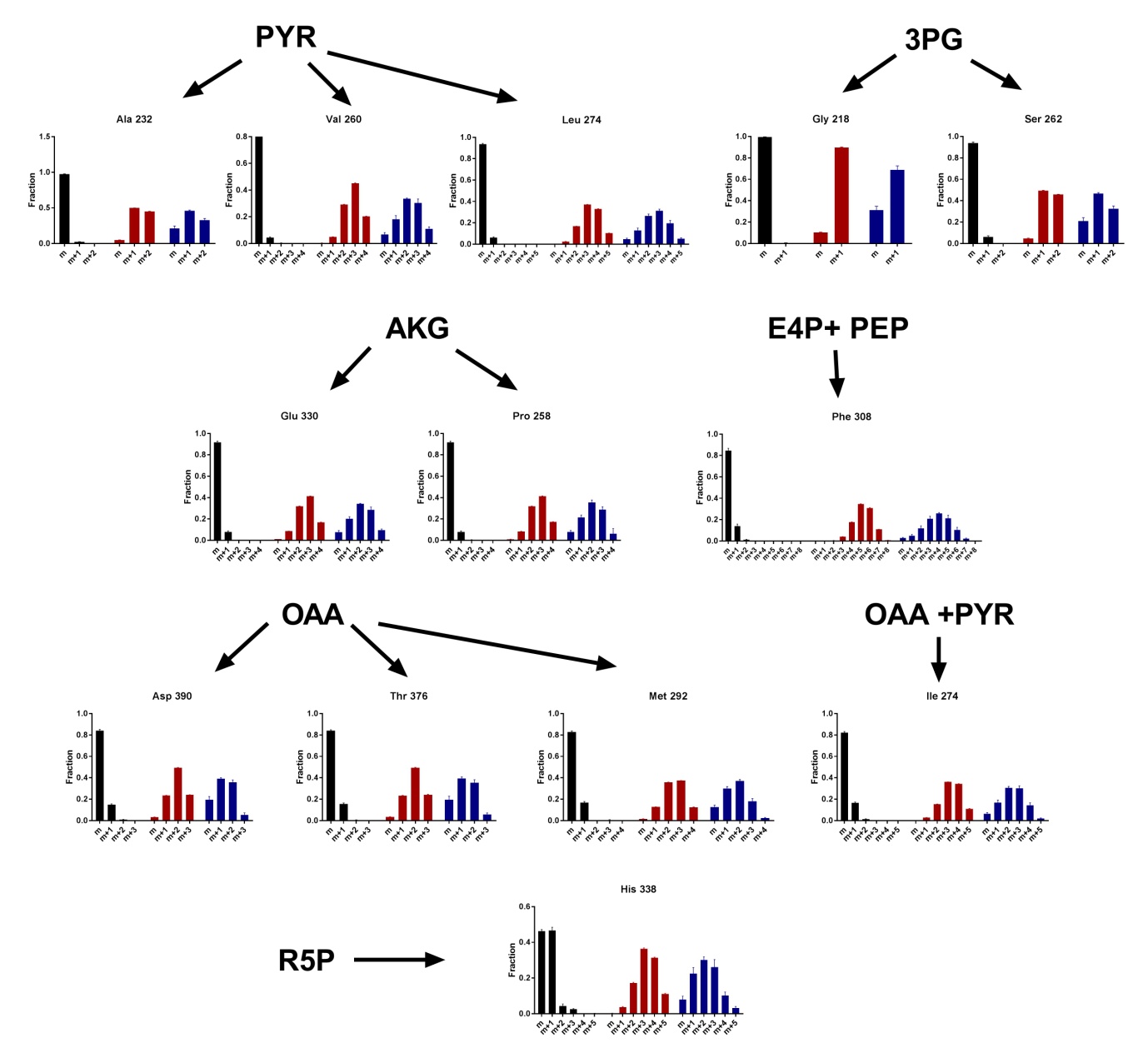


**Supplementary Figure S1.** Corrected MID values for [M-85] fragment ions of TBDMCS-derivatized amino acids in the isotopic steady state under heterotrophic growth with D-[1-^13^C]fructose (black bars) or [1,2-^13^C]glycerol (red bars) and under mixotrophic growth with [1,2-^13^C]glycerol and unlabelled CO_2_ (blue bars). The y-axis represent the number of ^13^C atoms incorporated into the amino acid molecule, increasing the m/z value by M+1, M+2 and so on. The MID of alanine, valine, serine, glycine, glutamate, proline, tyrosine, phenylalanine, aspartate, threonine, methionine, lysine, and histidine is mapped to their respective precursor molecules pyruvate (PYR), 3-phosphoglycerate (3-PG), alpha-ketoglutarate (AKG), erythrose-4phosphate (E4P), phosphoenolpyruvate (PEP), oxaloacetate (OAA) and ribose-5-phosphate (R5P).


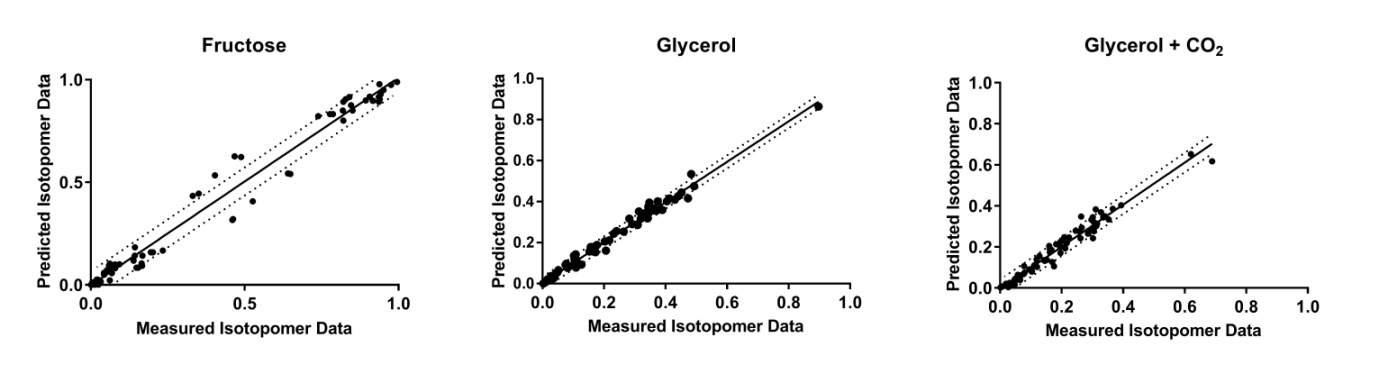


**Supplementary Figure S2**. Graph showing the correlation between experimentally measured amino acid MIDs and MID values predicted using OpenFLUX. The minimal residual error of the OpenFLUX prediction was reduced by removing outlier MIDs obtained from the correlation.

**Supplementary Table S1.** The list of oligonucleotide primers used for RT-PCR.

| **Gene^1^** | **Accession id** | **Reaction** |  | **Sequence** |  |  |
| --- | --- | --- | --- | --- | --- | --- |
| *tktA* | H16_A3147 | F6P + G3P = E4P + X5P | F | 5'- GCGTGAACCCGAAGACCAAC -3' | | |
|  |  |  | R | 5'- TCCTGCTTGTCGAACACGGT- 3' | | |
| *fbp* | H16_A0999 | F16P = F6P | F | 5' - ACCCGAAGGGCGAATACCTG -3' | | |
|  |  |  | R | 5' - TGCAGCACCGAGAAGATGGT -3' | | |
| *cbbL2* | H16_B1395 | RUBP + CO2 = 3PG + 3PG | F | 5' - GCGACATGTACCGTGCCAAG - 3' | | |
|  |  |  | R | 5' - ATGATCGACGCGGTCAGGTT -3' | | |
| *pyk1* | H16_A0567 | PEP = PYR | F | 5' - AAGCCGAGAAGTCCGAGGTG -3' | | |
|  |  |  | R | 5' - GCAATCGCCTTCACCTGCAA -3' | | |
| *glta* | H16_A2627 | ACCOA + OAA = CIT | F | 5' - AAGCTGATGCGCGAAACCTG -3' | | |
|  |  |  | R | 5' - TCGTCTTCCAGCGCGATCTT -3' | | |
| *icd3* | H16_B1016 | ISCIT = AKG + CO2 | F | 5' - GAATGGCCGGCGTCGAATAC -3' | | |
|  |  |  | R | 5' - GCACATTGACCGAGCGGAAG -3' | | |
| *mdh1* | H16_A2634 | MAL = OAA | F | 5' - AGGGTATGGAGCGCAAGGAC -3' | | |
|  |  |  | R | 5' - CATGGCGATGTAGGCGTTGG -3' | | |
| *pyc* | H16_A1251 | PYR + CO2 = OAA | F | 5' - GGAGATGGTCACCGGGATCG -3' | | |
|  |  |  | R | 5' - GCGTGACCGTTGAGCGAAAT -3' | | |
| *maeB* | H16_A1002 | MAL = CO2 + PYR | F | 5' - AAGGACGTGGCCAAGGTGAA -3' | | |
|  |  |  | R | 5' - CGCGGCCAACATAGATCACG -3' | | |
| *ppc* | H16_A2921 | PEP + CO2 = OAA | F | 5' - TGTCGGACCTGGTCGAAGTG -3' | | |
|  |  |  | R | 5' - GGTCTCGAACAGCGGGATCA -3' | | |
| *pepck* | H16_A3711 | OAA = PEP + CO2 | F | 5' - CGACGGCTCCCAGGAAGAAT -3' | | |
|  |  |  | R | 5' - CCGACAGTGCCAGGAAGGAA -3' | | |
| *iclA* | H16_A2211 | ISCIT = GLYOXY + SUC | F | 5' - CCCGTACTGATTCGCTGGGT -3' | | |
|  |  |  | R | 5' - TGACGTCACCATTGCCCAGT -3' | | |
| *pgi* | H16_A1501 | F6P = G6P | F | 5' - GCCATGGACGAGCATTTCCG -3' | | |
|  |  |  | R | 5' - CATGCAGCTGGTGGGCAAAT -3' | | |
| *pgam* | H16_A0493 | 3PG = 2PG | F | 5' - GGTCTATGCCAGCGACCTGT -3' | | |
|  |  |  | R | 5' - GGCTACTTCGGCGTAGGTCT -3' | | |
| *fba* | H16_A0568 | F16P = DHAP + G3P | F | 5' - TACGCCGGTGAGCATTTCCT -3' | | |
|  |  |  | R | 5' - GAGCCGTCCATCATCACCGA -3' | | |
| *eda* | H16_B1213 | KDPG = G3P + PYR | F | 5' - GGTGATCCCGGTGCTGGAA -3' | | |
|  |  |  | R | 5' - CTTGATCGCCTCCAGTGCCA -3' | | |
| *phaC1* | H16_A1437 |  | F | 5' - AGCGGCCAGGACAAGATCAA -3' | | |
|  |  |  | R | 5' - GTGTCGGCAAAGTCCAGCAG -3' | | |
| 16S |  |  | 16S-27F | 5' - AGAGTTTGATCCTGGCTCAG - 3' | | |
|  |  |  | 16S-533R | 5' - TTACCGCGGCTGCTGGCAC - 3' | | |

^1^Genes: *tktA* (transketolase); *fbp* (fructose bisphosphatase); *rbcL* (ribulose-1,5,bisphosphate carboyxylase/oxygenase, large subunit)); *pyk* (pyruvate kinase); *gltA* (citrate-synthase); *icd3* (isocitrate dehydrogenase); *mdh* (malate dehydrogenase); *pyc* (pyruvate carboxylase); *maeB* (malate decarboxylase), *ppc* (PEP carboxylase); *pck* (PEP carboxykinase); *icl* (isocitrate lyase); *pgi* (phosphoglucose isomerase); *pgmA* (phosphoglycerate mutase); *phaC1* (polyhydroxyalkanoate synthase), *eda* (2-keto-3-deoxyphosphogluconate aldolase); *fba* (fructose bisphosphate aldolase).

**Supplementary Table S2.** Metabolic model for central carbon metabolism with the carbon atom transition used as input for OpenFLUX analysis.

| R1 | GLY = DHAP | abc = abc |
| --- | --- | --- |
| R2 | F6P = G6P | abcdef = abcdef |
| R3 | G6P = F6P | abcdef = abcdef |
| R4 | F16P = F6P | abcdef = abcdef |
| R5 | F16P = DHAP + G3P | abcdef = cba + def |
| R6 | DHAP + G3P = F16P | cba + def = abcdef |
| R7 | DHAP = G3P | abc = abc |
| R8 | G3P = DHAP | abc = abc |
| R9 | G3P = 3PG | abc = abc |
| R10 | 3PG = G3P | abc = abc |
| R11 | 3PG = PEP | abc = abc |
| R12 | PEP = 3PG | abc = abc |
| R13 | PEP = PYR | abc = abc |
| R14 | PYR = PEP | abc = abc |
| R15 | PYR = ACCOA + CO2 | abc = bc + a |
| R16 | OAA + ACCOA = CIT | abcd + ef = dcbfea |
| R17 | CIT = ISCIT | abcdef = abcdef |
| R18 | ISCIT = CIT | abcdef = abcdef |
| R19 | ISCIT = AKG + CO2 | abcdef = abcde + f |
| R20 | AKG = 0.5 SUCOA + 0.5 SUCOA + CO2 | abcde = 0.5 bcde + 0.5 edcb + a |
| R21 | SUCOA = 0.5 SUC + 0.5 SUC | abcd = 0.5 abcd + 0.5 dcba |
| R22 | SUC = MAL | abcd = abcd |
| R23 | MAL = SUC | abcd = abcd |
| R24 | MAL = OAA | abcd = abcd |
| R25 | OAA = MAL | abcd = abcd |
| R26 | G6P = 6PG | abcdef = abcdef |
| R27 | 6PG = PYR + G3P | abcdef = abc + def |
| R28 | ISCIT = GLYOXY + 0.5 SUC + 0.5 SUC | abcdef = ab + 0.5 edcf + 0.5 fcde |
| R29 | GLYOXY + ACCOA = MAL | ab + cd = abdc |
| R30 | MAL = PYR + CO2 | abcd = abc + d |
| R31 | PEP + CO2 = OAA | abc + d = abcd |
| R32 | OAA = PEP + CO2 | abcd = abc + d |
| R33 | OAA = PYR + CO2 | abcd = abc + d |
| R34 | RU5P = R5P | abcde = abcde |
| R35 | R5P = RU5P | abcde = abcde |
| R36 | RU5P = X5P | abcde = abcde |
| R37 | X5P = RU5P | abcde = abcde |
| R38 | X5P + R5P = G3P + S7P | abcde + fghij = cde + abfghij |
| R39 | G3P + S7P = X5P + R5P | cde + abfghij = abcde + fghij |
| R40 | S7P + G3P = E4P + F6P | abcdefg + hij = defg + abchij |
| R41 | E4P + F6P = S7P + G3P | defg + abchij = abcdefg + hij |
| R42 | E4P + X5P = F6P + G3P | fghi + abcde = abfghi + cde |
| R43 | F6P + G3P = E4P + X5P | abfghi + cde = fghi + abcde |
| R44 | DHAP + E4P = S7P | efg + abcd = gfeabcd |
| R45 | RU5P = RUBP | abcde = abcde |
| R46 | RUBP + CO2 = 3PG + 3PG | abcde + f = fba + cde |
| R47 | AKG = GLU | abcde = abcde |
| R48 | AKG = PRO | abcde = abcde |
| R49 | AKG + CO2 = ARG | abcde + f = abcdef |
| R50 | OAA = ASP | abcd = abcd |
| R51 | PYR = ALA | abc = abc |
| R52 | 3PG = SER | abc = abc |
| R53 | 3PG = CYS | abc = abc |
| R54 | 3PG = GL + MTHF | abc = ab + c |
| R55 | OAA = GL + ACCOA | abcd = ab + cd |
| R56 | OAA = THR | abcd = abcd |
| R57 | OAA + MTHF = MET | abcd + e = abcde |
| R58 | OAA + PYR = LYS + CO2 | abcd + efg = bcdgfe + a |
| R59 | PYR + PYR = VAL + CO2 | abc + def = abcef + d |
| R60 | PYR + PYR = ISV + CO2 | abc + def = abefc + d |
| R61 | ISV + ACCOA = LEU + CO2 | abcde + fg = fgbcde + a |
| R62 | OAA + PYR = ILE + CO2 | abcd + efg = abfcdg + e |
| R63 | E4P + PEP = SHKM | abcd + efg = efgabcd |
| R64 | SHKM + PEP = CHRM | abcdefg + hij = abcdefghij |
| R65 | CHRM = PHE + CO2 | abcdefghij = hijbcdefg + a |
| R66 | CHRM = TYR + CO2 | abcdefghij = hijbcdefg + a |
| R67 | CHRM = ANTHR + G3P | abcdefghij = abcdefg + hij |
| R68 | ANTHR + R5P = CPADR5P | abcdefg + hijkl = abcdefghijkl |
| R69 | CPADR5P = INDG + CO2 | abcdefghijkl = abcdfghijkl + e |
| R70 | INDG = TRP | abcdfghijkl = abcdfghijkl |
| R71 | R5P + MTHF = HIS | abcde + f = edcbaf |
| R72 | ACCOA = PHB_B | |

**Supplementary Table S3.** 95% confidence interval estimates for the flux predictions of selected reactions.

| s.no | Reactions | Hetero-Fructose | Hetero- glycerol | Mixo-glycerol |
| --- | --- | --- | --- | --- |
| 1. | G3P ↔ 3PG | (0.90,1.08) | (-1.05, -0.94) | (-5.84,-5.08) |
| 2. | 3PG ↔ PEP | (0.80, 0.96) | (-0.3,-0.06) | (-0.03,0.0) |
| 3. | PYR → ACCOA + CO2 | (0.38,0.50) | (0.009, 0.15) | (1.09,1.3) |
| 4. | OAA + ACCOA → CIT | (0.39, 0.6) | (0.01,0.04) | (0.9,1.1) |
| 5. | ISCIT → AKG + CO2 | (0.38,0.45) | (0.007,0.01) | (0.75, 1.0) |
| 6. | 6PG → PYR + G3P | (0.96,1.27) | (0.87,0.96) | (1.71,2.13) |
| 7. | ISCIT → GLYOXY + 0.5 SUC + 0.5 SUC | (0.0,0.0) | (0.0,0.001) | (0.22,0.32) |
| 8. | PEP + CO2 ↔ OAA | (1.26, 2.36) | (0.001,0.09) | (0.88,1.01) |
| 9. | OAA → PYR + CO2 | (1.18,2.77) | (0.0,0.003) | (0.27,0.44) |
| 10. | F6P + G3P ↔ E4P + X5P | (0.0,0.03) | (0.32,0.35) | (0.9, 0.99) |
| 11. | RUBP + CO2 → 3PG + 3PG | (0.0,0.03) | (0.2, 0.44) | (2.7, 2.96) |

**Supplementary Table S4.** Mass Isotopomer Distribution of amino acids under heterotrophic growth with D-[1-^13^C]fructose.

| Amino Acid |  | m/z | M | M+1 | M+2 | M+3 | M+4 | M+5 | M+6 | M+7 | M+8 | M+9 |
| --- | --- | --- | --- | --- | --- | --- | --- | --- | --- | --- | --- | --- |
| Alanine | [M-85] | 232 | 0.9759 ± 0.0027 | 0.0230 ± 0.0035 | 0.0011 ± 0.0009 | 0 | 0 | 0 | 0 | 0 | 0 | 0 |
|  | [M-57] | 260 | 0.3509 ± 0.0135 | 0.6407 ± 0.0138 | 0.0078 ± 0.0003 | 0.0006 ± 0.0006 | 0 | 0 | 0 | 0 | 0 | 0 |
| Glycine | [M-85] | 218 | 0.9950 ± 0.0021 | 0.0049 ± 0.0021 | 0 | 0 | 0 | 0 | 0 | 0 | 0 | 0 |
|  | [M-57] | 246 | 0.9357 ± 0.0068 | 0.0638 ± 0.0072 | 0.0005 ± 0 |  | 0 | 0 | 0 | 0 | 0 | 0 |
| Valine | [M-85] | 260 | 0.9513 ± 0.0058 | 0.0436 ± 0.0057 | 0.0049 ± 0.0005 | 0 | 0.00003 ± 0 | 0 | 0 | 0 | 0 | 0 |
|  | [M-57] | 288 | 0.3314 ± 0.0117 | 0.6486 ± 0.0148 | 0.0194 ± 0.0028 | 0.0006 ± 0 | 0.00002 ± 0 | 0.00001 ± 0 | 0 | 0 | 0 | 0 |
| Leucine | [M-85] | 274 | 0.9364 ± 0.0068 | 0.0620 ± 0.0067 | 0.0013 ± 0.0002 | 0.0002 + 0 | 0.00001 ± 0 | 0 | 0 | 0 | 0 | 0 |
| Isoleucine | [M-85] | 274 | 0.8210975 ± 0.0120 | 0.1654325 ± 0.0083 | 0.01305 ± 0.0040 | 0.0003375 ± 0 | 0 | 0 | 0 | 0 | 0 | 0 |
| Methionine | [M-85] | 292 | 0.8280 ± 0.0094 | 0.1671 ± 0.0112 | 0 | 0 | 0 | 0 | 0 | 0 | 0 | 0 |
|  | [M-57] | 320 | 0.7391 ± 0.0183 | 0.2339 ± 0.0158 | 0.0239 ± 0.0044 | 0.0029 ± 0 | 0 | 0 | 0 | 0 | 0 | 0 |
| Serine | [M-85] | 362 | 0.9378 ± 0.0109 | 0.0619 ± 0.0105 | 0.0003 ± 0 | 0 | 0 | 0 | 0 | 0 | 0 | 0 |
|  | [M-57] | 390 | 0.9064 ± 0.0159 | 0.0789 ± 0.0155 | 0.0146 ± 0.0014 | 0 | 0 | 0 | 0 | 0 | 0 | 0 |
| Threonine | [M-85] | 376 | 0.8401 ± 0.0089 | 0.1544 ± 0.0098 | 0.0055 ± 0.0016 | 0 | 0 | 0 | 0 | 0 | 0 | 0 |
|  | [M-57] | 404 | 0.7867 ± 0.0107 | 0.1954 ± 0.0125 | 0.0161 ± 0.0056 | 0.0017 ± 0 | 0 | 0 | 0 | 0 | 0 | 0 |
| Phenylalanine |  | 302 | 0.9390 ± 0.0068 | 0.0590 ± 0.0069 | 0.00195 ± 0.008 | 0 | 0 | 0 | 0 | 0 | 0 | 0 |
|  | [M-85] | 308 | 0.8456 ± 0.0215 | 0.1394 ± 0.0191 | 0.0125 ± 0.0037 | 0.0022 ± 0.0001 | 0.0002 ± 0 | 0 | 0 | 0 | 0 | 0 |
|  | [M-57] | 336 | 0.8209 ± 0.0256 | 0.1435 ± 0.0182 | 0.0327 ± 0.0082 | 0.0028 ± 0 | 0 | 0 | 0 | 0 | 0 | 0 |
| Aspartic acid |  | 302 | 0.8938 ± 0.0062 | 0.0929 ± 0.0029 | 0.0132 ± 0.0035 | 0 | 0 | 0 | 0 | 0 | 0 | 0 |
|  | [M-85] | 390 | 0.8415 ± 0.0102 | 0.1486 ± 0.0085 | 0.0094 ± 0.0017 | 0.0004 ± 0 | 0 | 0 | 0 | 0 | 0 | 0 |
|  | [M-57] | 418 | 0.7777 ± 0.0107 | 0.2021 ±0.0077 | 0.0189 ± 0.0023 | 0.0011 ± 0 | 0.0002 ± 0 | 0 | 0 | 0 | 0 | 0 |
| Glutamic acid | [M-85] | 330 | 0.9176 ± 0.0118 | 0.0784 ± 0.0112 | 0.0031 ±.0006 | 0.0008 ± 0 | 0.00005 ± 0 | 0 | 0 | 0 | 0 | 0 |
|  | [M-57] | 432 | 0.8192 ± 0.0129 | 0.1679 ± 0.0102 | 0.0125 ± .0028 | 0.0002 ± 0 | 0.0001 ±0 | 0 | 0 | 0 | 0 | 0 |
| Histidine | [M-85] | 338 | 0.4626 ± 0.0091 | 0.4672 ± 0.0173 | 0.0427 ± 0.0119 | 0.0255 ± 0.0030 | 0.0008 ± 0 | 0.0009 ± 0 | 0 | 0 | 0 | 0 |
|  | [M-57] | 440 | 0.4600 ± 0.0100 | 0.4884 ± 0.0228 | 0.0432 ± 0.0130 | 0.0082 ±0.0039 | 0 | 0 | 0 | 0 | 0 | 0 |

**Supplementary Table S5.** Mass Isotopomer Distribution of amino acids under heterotrophic growth with [1,2-^13^C]glycerol.

| Amino Acid |  | m/z | M | M+1 | M+2 | M+3 | M+4 | M+5 | M+6 | M+7 | M+8 | M+9 |
| --- | --- | --- | --- | --- | --- | --- | --- | --- | --- | --- | --- | --- |
| Alanine | [M-85] | 232 | 0.0495 ± 0.0022 | 0.5002 ± 0.0009 | 0.4504 ± 0.0023 | 0 | 0 | 0 | 0 | 0 | 0 | 0 |
|  | [M-57] | 260 | 0.0221 ± 0.0013 | 0.1317 ± 0.0056 | 0.7290 ± 0.0131 | 0.117145 ± 0.0070 | 0 | 0 | 0 | 0 | 0 | 0 |
| Glycine | [M-85] | 218 | 0.1025 ± 0.0052 | 0.8974 ± 0.0052 | 0 | 0 | 0 | 0 | 0 | 0 | 0 | 0 |
|  | [M-57] | 246 | 0.0432 ± 0.0025 | 0.4732 ± 0.0035 | 0.4835 ± 0.0041 | 0 | 0 | 0 | 0 | 0 | 0 | 0 |
| Valine | [M-85] | 260 | 0.0046 ± 0.0001 | 0.0488 ± 0.0022 | 0.2918 ± 0.0021 | 0.4519 ± 0.0025 | 0.2029 ± 0.0024 | 0 | 0 | 0 | 0 | 0 |
|  | [M-57] | 288 | 0.0029 ± 0 | 0.0169 ± 0.0011 | 0.1079 ± 0.0040 | 0.4304 ± 0.0026 | 0.3897 ± 0.0045 | 0.0521 ± 0.0023 | 0 | 0 | 0 | 0 |
| Leucine | [M-85] | 274 | 0.0022 ± 0.0001 | 0.0248 ± 0.0011 | 0.1678 ± 0.0012 | 0.3708 ± 0.0010 | 0.3302 ± 0.0012 | 0.1041 ± 0.0005 | 0 | 0 | 0 | 0 |
| Isoleucine | [M-85] | 274 | 0.0039 ± 0.0001 | 0.0279 ± 0.0006 | 0.1532 ± 0.0024 | 0.3613 ± 0.0011 | 0.3435 ± 0.0008 | 0.1103 ± 0.0035 | 0 | 0 | 0 | 0 |
| Methionine | [M-85] | 292 | 0.0152 ± 0.0016 | 0.1277 ± 0.0009 | 0.3585 ± 0.0012 | 0.3735 ± 0.0012 | 0.1249 ± 0.0022 | 0 | 0 | 0 | 0 | 0 |
|  | [M-57] | 320 | 0.0066 ± 0.0002 | 0.0418 ± 0.0018 | 0.2062 ± 0.0012 | 0.3885 ± 0.0032 | 0.2921 ± 0.0024 | 0.0647 ± 0.0032 | 0 | 0 | 0 | 0 |
| Serine | [M-85] | 362 | 0.0474 ± 0.0027 | 0.4949 ± 0.0019 | 0.4577 ± 0.0027 | 0 | 0 | 0 | 0 | 0 | 0 | 0 |
|  | [M-57] | 390 | 0.0217 ± 0.0013 | 0.1540 ± 0.0086 | 0.6809 ± 0.0186 | 0.1434 ± 0.0093 | 0 | 0 | 0 | 0 | 0 | 0 |
| Threonine | [M-85] | 376 | 0.0342 ± 0.0017 | 0.2322 ± 0.0028 | 0.4928 ± 0.0048 | 0.2407 ± 0.0066 | 0 | 0 | 0 | 0 | 0 | 0 |
|  | [M-57] | 404 | 0.0100 ± 0.0003 | 0.0777 ± 0.0017 | 0.3444 ± 0.0029 | 0.4406 ± 0.0048 | 0.1272 ± 0.0024 | 0 | 0 | 0 | 0 | 0 |
| Phenylalanine |  | 302 | 0.0428 ± 0.0011 | 0.4737 ± 0.004 | 0.4834 ± 0.0038 | 0 | 0 | 0 | 0 | 0 | 0 | 0 |
|  | [M-85] | 308 | 0.0029 ± 0.0002 | 0.0001 ± 0 | 0.0056 ± 0.0005 | 0.0405 ± 0.0019 | 0.1772 ± 0.0016 | 0.3472 ± 0.0015 | 0.3098 ± 0.0032 | 0.1092 ± 0.0009 | 0.0073 ± 0.0008 | 0 |
|  | [M-57] | 336 | 0.0004 ± 0 | 0 | 0.0027 ± 0.0003 | 0.0171 ± 0.0017 | 0.0821 ± 0.0027 | 0.2639 ± 0.0026 | 0.3745 ± 0.0058 | 0.2175 ± 0.0025 | 0.0396 ± 0.0036 | 0.0020 ± 0.0004 |
| Aspartic acid |  | 302 | 0.0879 ± 0.002 | 0.4883 ± 0.0007 | 0.4238 ± 0.0021 | 0 | 0 | 0 | 0 | 0 | 0 | 0 |
|  | [M-85] | 390 | 0.0326 ± 0.0008 | 0.2335 ± 0.0029 | 0.4937 ± 0.0021 | 0.2401 ± 0.0018 | 0 | 0 | 0 | 0 | 0 | 0 |
|  | [M-57] | 418 | 0.0130 ± 0.0007 | 0.0827 ± 0.0018 | 0.3460 ± 0.0031 | 0.4339 ± 0.0033 | 0.1243 ± 0.0014 | 0 | 0 | 0 | 0 | 0 |
| Glutamic acid | [M-85] | 330 | 0.0113 ± 0.0003 | 0.0865 ± 0.0007 | 0.3197 ± 0.0015 | 0.4128 ± 0.0016 | 0.1697 ± 0.0005 | 0 | 0 | 0 | 0 | 0 |
|  | [M-57] | 432 | 0.0037 ± 0.0004 | 0.0279 ± 0.0003 | 0.1568 ± 0.0017 | 0.3639 ± 0.0013 | 0.3418 ± 0.0019 | 0.1059 ± 0.0013 | 0 | 0 | 0 | 0 |
| Histidine | [M-85] | 338 | 0.0018 ± 0.0001 | 0.0368 ± 0.0030 | 0.1722 ± 0.0042 | 0.3640 ± 0.0047 | 0.3136 ± 0.0037 | 0.1114 ± 0.0027 | 0 | 0 | 0 | 0 |
|  | [M-57] | 440 | 0.0016 ± 0.0012 | 0.0137 ± 0.0021 | 0.0754 ± 0.0036 | 0.2825 ± 0.0029 | 0.4047 ± 0.0087 | 0.2061 ± 0.0014 | 0.0158 ± 0.0035 | 0 | 0 | 0 |

**Supplementary Table S6.** Mass Isotopomer Distribution of amino acids under mixotrophic growth with [1,2-^13^C]glycerol and CO_2_.

| Amino Acid |  | m/z | M | M+1 | M+2 | M+3 | M+4 | M+5 | M+6 | M+7 | M+8 | M+9 |
| --- | --- | --- | --- | --- | --- | --- | --- | --- | --- | --- | --- | --- |
| Alanine | [M-85] | 232 | 0.2128 ± 0.0341 | 0.4596 ± 0.0103 | 0.3275 ± 0.0239 | 0 | 0 | 0 | 0 | 0 | 0 | 0 |
|  | [M-57] | 260 | 0.1996 ± 0.0337 | 0.3592 ± 0.0214 | 0.4174 ± 0.0544 | 0.0238 ± 0.0124 | 0 | 0 | 0 | 0 | 0 | 0 |
| Glycine | [M-85] | 218 | 0.3111 ± 0.0365 | 0.6889 ± 0.0365 | 0 | 0 | 0 | 0 | 0 | 0 | 0 | 0 |
|  | [M-57] | 246 | 0.3019 ± 0.0322 | 0.6202 ± 0.0331 | 0.0778 ± 0.0263 | 0 | 0 | 0 | 0 | 0 | 0 | 0 |
| Valine | [M-85] | 260 | 0.0680 ± 0.0141 | 0.1824 ± 0.0264 | 0.3352 ± 0.0050 | 0.3045 ± 0.0294 | 0.1097 ± 0.0156 | 0 | 0 | 0 | 0 | 0 |
|  | [M-57] | 288 | 0.0652 ± 0.0132 | 0.1620 ± 0.0281 | 0.2968 ± 0.0182 | 0.3150 ± 0.0292 | 0.1526 ± 0.0299 | 0.0083 ± 0.0003 | 0 | 0 | 0 | 0 |
| Leucine | [M-85] | 274 | 0.0467 ± 0.0098 | 0.1282 ± 0.0227 | 0.2652 ± 0.0169 | 0.3132 ± 0.0143 | 0.1966 ± 0.0253 | 0.0501 ± 0.0093 | 0 | 0 | 0 | 0 |
| Isoleucine | [M-85] | 274 | 0.0630 ± 0.0123 | 0.1669 ± 0.0247 | 0.3057 ± 0.0141 | 0.3023 ± 0.0226 | 0.1426 ± 0.0239 | 0.0194 ± 0.0052 | 0 | 0 | 0 | 0 |
| Methionine | [M-85] | 292 | 0.1252 ± 0.0184 | 0.2987 ± 0.0191 | 0.3709 ± 0.0131 | 0.1813 ± 0.0240 | 0.0238 ± 0.0059 | 0 | 0 | 0 | 0 | 0 |
|  | [M-57] | 320 | 0.1144 ± 0.0203 | 0.2522 ± 0.0259 | 0.3458 ± 0.0147 | 0.2137 ± 0.0306 | 0.0634 ± 0.0147 | 0.0105 ± 0.0009 | 0 | 0 | 0 | 0 |
| Serine | [M-85] | 362 | 0.2083 ± 0.0329 | 0.4664 ± 0.0091 | 0.3252 ± 0.0249 | 0 | 0 | 0 | 0 | 0 | 0 | 0 |
|  | [M-57] | 390 | 0.2044 ± 0.0301 | 0.3962 ± 0.0085 | 0.3813 ± 0.0359 | 0.0180 ± 0.0016 | 0 | 0 | 0 | 0 | 0 | 0 |
| Threonine | [M-85] | 376 | 0.1956 ± 0.0318 | 0.3937 ± 0.0144 | 0.3530 ± 0.0287 | 0.0576 ± 0.0137 | 0 | 0 | 0 | 0 | 0 | 0 |
|  | [M-57] | 404 | 0.1695 ± 0.0243 | 0.3279 ± 0.0245 | 0.3661 ± 0.0266 | 0.1113 ± 0.0236 | 0.0250 ± 0.0009 | 0 | 0 | 0 | 0 | 0 |
| Phenylalanine |  | 302 | 0.2943 ± 0.0338 | 0.6091 ± 0.0236 | 0.0965 ± 0.0242 | 0 | 0 | 0 | 0 | 0 | 0 | 0 |
|  | [M-85] | 308 | 0.0279 ± 0.0061 | 0.0491 ± 0.0130 | 0.1184 ± 0.0245 | 0.2083 ± 0.0237 | 0.2584 ± 0.0085 | 0.2121 ± 0.0303 | 0.1038 ± 0.0242 | 0.0213 ± 0.0066 | 0.0004 ± 0 | 0 |
|  | [M-57] | 336 | 0.0268 ± 0.0062 | 0.0480 ± 0.0130 | 0.1133 ± 0.0233 | 0.1965 ± 0.0244 | 0.2459 ± 0.0120 | 0.2168 ± 0.0282 | 0.1202 ± 0.0307 | 0.0307 ± 0.0099 | 0.0015 ± 0.0002 | 0.0002 ± 0 |
| Aspartic acid |  | 302 | 0.3550 ± 0.0410 | 0.5093 ± 0.0131 | 0.1357 ± 0.0307 | 0 | 0 | 0 | 0 | 0 | 0 | 0 |
|  | [M-85] | 390 | 0.1958 ± 0.0287 | 0.3921 ± 0.0086 | 0.3582 ± 0.0218 | 0.0539 ± 0.0185 | 0 | 0 | 0 | 0 | 0 | 0 |
|  | [M-57] | 418 | 0.1767 ± 0.0303 | 0.3209 ± 0.0211 | 0.3703 ± 0.0257 | 0.1034 ± 0.0311 | 0.0286 ± 0.0075 | 0 | 0 | 0 | 0 | 0 |
| Glutamic acid | [M-85] | 330 | 0.0769 ± 0.0149 | 0.2009 ± 0.0203 | 0.3411 ± 0.0046 | 0.2860 ± 0.0248 | 0.0950 ± 0.0112 | 0 | 0 | 0 | 0 | 0 |
|  | [M-57] | 432 | 0.0644 ± 0.0204 | 0.1618 ± 0.0302 | 0.3047 ± 0.0222 | 0.3044 ± 0.0255 | 0.1451 ± 0.0376 | 0.0196 ± 0.0011 | 0 | 0 | 0 | 0 |
| Histidine | [M-85] | 338 | 0.0797 ± 0.0183 | 0.2241 ± 0.0347 | 0.3013 ± 0.0174 | 0.2613 ± 0.0420 | 0.1017 ± 0.0200 | 0.0319 ± 0.0087 | 0 | 0 | 0 | 0 |
|  | [M-57] | 440 | 0.0634 ± 0.0149 | 0.1595 ± 0.0277 | 0.2636 ± 0.0223 | 0.2882 ± 0.0203 | 0.1752 ± 0.0333 | 0.0473 ± 0.0118 | 0.0027 ± 0.0032 | 0 | 0 | 0 |
